# Field study on the utility of fluid obtained from testicles as a sample for detecting antibodies to selected swine pathogens

**DOI:** 10.1038/s41598-025-05380-8

**Published:** 2025-07-01

**Authors:** Agata Augustyniak, Ewelina Czyżewska-Dors, Małgorzata Pomorska-Mól

**Affiliations:** 1 Department of Preclinical Sciences and Infectious Diseases, Poznan University of Life Sciences, Wołyńska 35, 60-637 Poznań, Poland; 2 Department of Internal Diseases and Diagnostics, Poznan University of Life Sciences, Wołyńska 35, 60-637 Poznań, Poland

**Keywords:** Processing fluid, Antibodies, Detection, ELISA, Immunology, Microbiology, Diseases

## Abstract

Processing fluid is a promising alternative to blood for monitoring porcine diseases, although certain aspects of its routine use remain unclear. This study evaluated serum from females and males, along with corresponding testicular only processing fluid, for antibodies against *Actionbacillus pleuropneumonie*, hepatitis E virus, porcine epidemic diarrhea virus, influenza A virus, *Erysipetothrix rhusiopathie and Mycoplasma hyopneumoniae*, using commercial ELISAs (ID Screen APP, Hepatitis E, PEDV, Influenza A from ID Vet, France; Civtest suis SE/MR from Hipra, Spain; and Mycoplasma hyopneumoniae from Idexx, USA). Differences in the proportion of positive results across sample types were analysed to assess the utility of testis-derived processing fluid for litter-level health monitoring. ROC analysis was used to establish optimal cut-offs for processing fluid, followed by evaluation of diagnostic performance using both manufacturer-recommended and ROC-derived thresholds. A pooling simulation was also performed. Results indicate that processing fluid collected exclusively from testes can detect antibodies against selected pathogens effectively. Some ELISA kits validated for serum may be applicable to processing fluid, provided that appropriate cut-off values are determined for this sample type. However, pooling processing fluid samples may reduce sensitivity and increase the risk of false-negative results. These findings highlight the potential of testis-derived processing fluid for large-scale serological surveillance while underscoring the need for test-specific validation.

## Introduction

Swine health is one of the most important factors influencing the performance of pig husbandry. The intensification of swine production during the last decades has increased the significance of multifactorial diseases^1,2^. In addition, within the last few years, difficult-to-control porcine diseases, like African swine fever, have emerged or re-emerged^3^. This complicates herd health management since efficient control of such diseases is often challenging and requires a complex approach. Among the most important strategies of herd health management is farm biosecurity. It includes all efforts to minimise the pathogen introduction and spread within the farm^3^. Biosecurity involves many aspects of farm management, like quarantine and acclimatisation, as the arrival of new pigs creates the highest possibility of pathogen introduction^3^. During quarantine, pigs are usually tested for various pathogens or specific antibodies, increasing farm biosecurity. Implementing control or eradication programs is another strategy that increases the herd’s health status; applying surveillance is crucial for monitoring its progress. Other approaches include, among others, the use of drugs and vaccines. Due to the associated costs, decisions regarding these interventions should be made with caution; laboratory analyses may support the decision-making process^4^. Consequently, porcine health management relies primarily on regular health status monitoring using direct or indirect laboratory diagnostic methods. One of the most commonly applied approaches is the analysis of specific antibodies against pathogens of interest to assess herd health status. For this purpose, ELISA tests are among the most widely used methods. They are highly cost-effective and allow for large-scale sample testing. ELISA assays are particularly valuable in naïve populations or when the disease status is unknown. In contrast to antibody detection, direct pathogen detection is often more challenging, as antibodies usually persist longer in the host than the pathogen itself. The window of opportunity for direct detection methods, such as PCR, is limited and depends on the stage of infection. ELISA facilitates broader epidemiological assessments by assessing the proportion of seropositive individuals or monitoring changes in antibody levels over time. Numerous commercial ELISA kits are available to detect specific antibodies in porcine serum.

Processing fluid (PF) consists of blood and tissue fluid recovered from castration and tail docking, usually performed between 3 and 5 days of piglet life. These procedures are still routinely performed on many farms worldwide. Therefore, PF’s collection does not require additional work and time and does not generate additional cost or stress for animals. Due to these reasons, PF has gained scientists’ attention as a new, possible alternative to blood, the most common sample type for diagnosing and monitoring pigs’ health status. The search for alternative sample types stems from challenges associated with blood collection and increasing emphasis on animal welfare. Blood collection is a labour- and time-intensive procedure that requires restraining the pigs, poses risks to both animals and personnel and negatively affects animal welfare^5,6^. Examples of already described alternative matrices include oral fluid or meat juice (MJ), which proved useful for important porcine pathogens antibodies and/or genetic material detection. Regarding PF, many aspects of its potential diagnostic utility remain unknown. Numerous studies confirmed the presence of porcine reproductive and respiratory syndrome virus (PRRSV) genetic material in the PF samples tested via PCR, and available data indicate its potential usefulness in improving the monitoring of this pathogen in breeding herds^7,8^. The feasibility of using PF for direct diagnostics by PCR has also been confirmed for *Mycoplasma (M.) hyopneumoniae* and porcine circovirus type 2^9,10^. Except for the pathogens’ genetic material, PF could also be used to assess the presence of piglets’ immunological indices by ELISA assay, including antibodies, cytokines, and acute phase proteins, representing a promising alternative to blood in assessing piglets’ immune status at processing age^11^. Antibodies present in the blood of newborn piglets are of colostral origin, representing circulating maternal antibodies, and are not proof of piglets’ infection or vaccination^12^. Therefore, antibodies contained in the PF may provide insight into indirect sow herd surveillance^12^. The data indicate that PF represents a sample that can be used to detect specific antibodies against various porcine pathogens^8,10,13,14^. It is worth noting that in previous studies, ELISA kits validated for serum were used to detect antibodies in PF. Until commercial ELISA kits validated for new matrices, including PF, become available, it is necessary to use ELISA kits validated for serum when testing PF.

The present study aimed to evaluate the diagnostic utility of testicular-only processing fluid (tPF) for detecting antibodies against selected three bacterial and three viral porcine pathogens: *Actinobacillus pleuropneumoniae* (*A.plueuropneumoniae*), *Erysipelothrix rhusiopathiae* (*E. rhusiopathiae*), hepatitis E virus (HEV), porcine epidemic diarrhoea virus (PEDV), influenza A virus (IAV), and *Mycoplasma hyopneumoniae*, using commercially available ELISA kits validated for serum samples. Previous studies assessing processing fluid or diagnostic purposes typically analysed mixed PF samples containing fluid from both testicles and tails. However, recent findings by Gomes-Neves et al. (2024) showed that only 22% of 15,683 weaners examined had docked tails^15^, reflecting a broader downward trend in tail docking, also observed in Poland. Therefore, this study focused specifically on evaluating the diagnostic performance of tPF. Additionally, results obtained from tPF were compared with those from serum samples collected from male and female piglets. Under field conditions, PF is typically tested as a pooled sample, with tissues from multiple litters combined in one container. However, pooling may affect diagnostic sensitivity by diluting target analytes, particularly in cases of low pathogen or antibody prevalence. Due to the limited data on this topic, the present study also investigated the effects of sample pooling and initial antibody levels on ELISA test outcomes.

## Results

### *A. pleuropneumoniae*

**Fig. 1:**
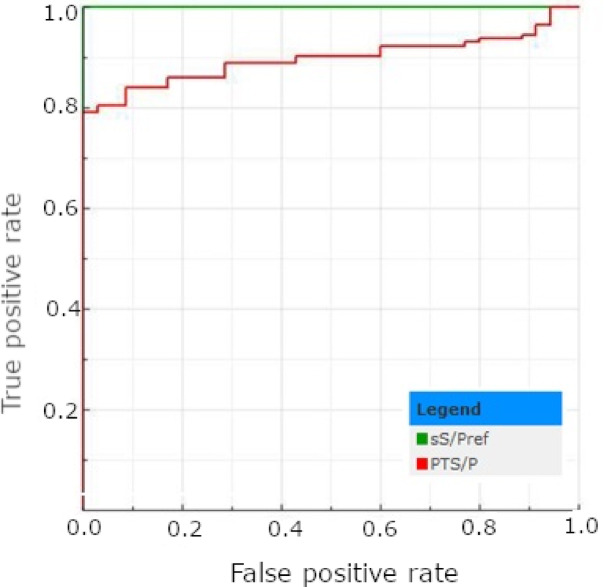
The ROC plots determined for the *A.pleuropneumoniae* ELISA test results obtained for serum (sS/Pref) and tPF (PTS/P).

Using the cut-off recommended by the ELISA test manufacturer (S/P%>27%), 93 out of 178 (52.25%) tPF samples and 143 out of 178 (80.34%) corresponding male serum samples were classified as positive (Table 1). No significant differences have been found between the number of positive results obtained from the serum of males and females (*p* > 0.05; Table 1). The optimal cut-off for tPF samples has been established based on Receiver Operating Characteristic (ROC) curve results at S/P%>15%. Using this value, 122 out of 178 (68.54%) tPT samples were classified as positive (Table 1). However, the statistically significant difference in positive sample proportion between piglets’ serum of both genders and tPF was observed, independently of the cut-off used (*p* < 0.05; Table 1). The descriptive statistics regarding the test’s results are presented in Table 2. The AUC value determined for tPF (0.897) was significantly lower when compared to the reference and was classified as considerable (*p* < 0.05; Fig. 1). Implementing a new cut-off improves the test’s sensitivity, NPV, and accuracy, although it slightly reduces specificity and PPV. It also increased the kappa coefficient (from 0.42 to 0.55); however, the agreement remained moderate. A detailed comparison of the selected test parameters and agreement measures depending on the implemented cut-off values is presented in Table 3.

**Table 1:** The comparison of anti-*A. Pleuropneumoniae* antibody presence in different matrices.

Sample type	Positive/Total	Proportion of positive samples (%)	95%CI
Male piglets’ serum	143/178	80.34^a^	73.88–85.51
Female piglets’ serum	132/159	83.02^a^	76.42–88.06
tPF (manufacturer’s cut-off)	93/178	52.25^b^	44.94–59.46
tPF (optimal cut-off)	122/178	68.54^c^	61.39–74.91

^a,b,c^Different letters represent a statistically significant difference between the analysed samples (*p* < 0.05); 95% CI − 95% confidence interval.

**Table 2:** Mean (± SD) and range (minimum and maximum) S/P% values obtained for *A. pleuropneumoniae* ELISA for various samples.

	Mean	Min.	Max	SD
Male piglets’ serum S/P%	159.16	6.19	549.42	107.23
Female piglets’ serum S/P%	159.73	10.34	370.49	95.52
tPF S/P%	53.60	3.10	284.12	51.02

Min. – minimum value; Max. – maximum value; SD – standard deviation.

**Table 3:** The comparison of the selected *A. pleuropneumoniae* test parameters and measures of agreement depending on the implemented cut-off values.

Cut-off (S/*P*%)	27	15
Sensitivity	0.65	0.82
Specificity	1	0.86
Positive predictive values	1	0.96
Negative predictive values	0.41	0.54
Accuracy	0.72	0.83
Kappa coefficient	0.42	0.55

In the next step, tPF pools were tested to determine the maximum dilution to detect one positive sample in a pool of negatives. Using the cut-off value recommended by the manufacturer, anti-*A. pleuropneumoniae* antibodies were detected only in 1 out of three low-positive samples at dilutions of 1:10. Nevertheless, using the ROC-calculated cut-off, all samples at dilution up to 1:20 and two out of three at dilution 1:40 were classified as positive. In general, 8% (1/12) and 66% (8/12) of low-positive samples were correctly classified as positive after applying the manufacturer’s and ROC-calculated cut-off, respectively. Using the manufacturer’s cut-off, all moderate-positive samples were correctly classified at dilution 1:10, and one out of three samples at dilution 1:20. Using ROC-calculated cut-off, all moderate-positive samples were correctly classified at dilutions up to 1:40. In total, 33% (4/12) and 75% (9/12) of moderate-positive samples were correctly classified as positive after applying manufacturer’s and ROC-calculated cut-off, respectively. Using the cut-off value recommended by the manufacturer regarding high-positive samples, antibodies were detected in all samples diluted 1:10 and in 1 out of three at dilutions 1:20. When the ROC-calculated cut-off was applied, all samples at dilution up to 1:40 and two out of three at dilution 1:80 were classified as positive. In general, 33% (4/12) and 92% (11/12) of high-positive samples were correctly classified as positive after applying the manufacturer’s and ROC-calculated cut-off, respectively.

### *E. rhusiopathiae*

**Fig. 2:**
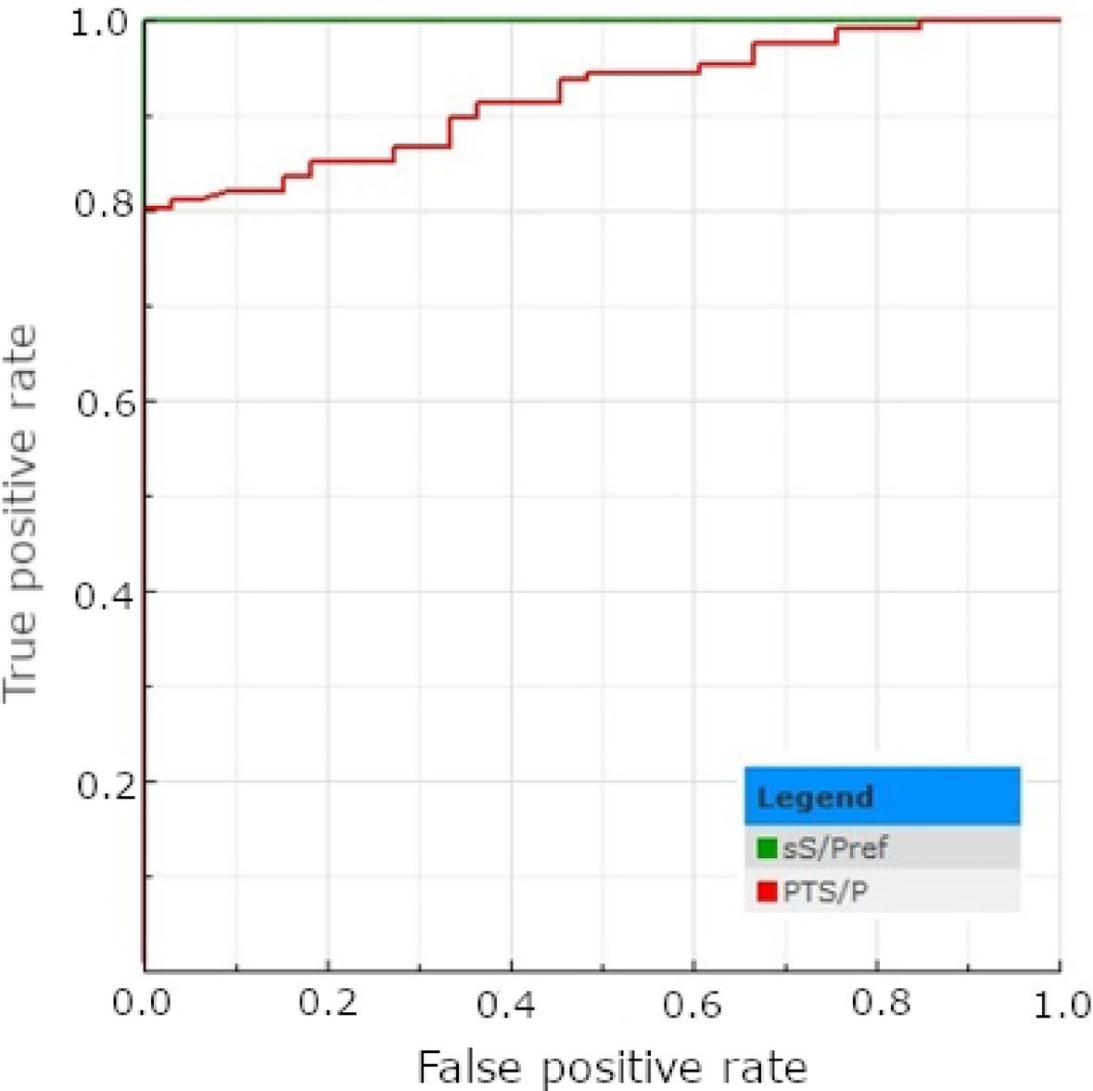
The ROC plots determined for the *E. rhusiopathiae* ELISA test results obtained for serum (sS/Pref) and tPF (PTS/P).

Interpreting the results following the cut-off recommended by the ELISA kit manufacturer for serum (40 IRPC), 89 out of 160 (55.62%) tested tPF samples were classified as positive for *E. rhusiopathiae* antibodies (Table 4). Among the corresponding male serum samples, 127 (79.38%) gave positive results (Table 4). No significant differences have been found between the number of positive results obtained from the serum of males and females (*p* > 0.05; Table 4). When using the cut-off recommended for serum, the number of serum samples positive for *E. rhusiopathiae* antibodies was significantly higher in both genders than in the tPF results (*p* < 0.05). The ROC curve analyses determined an optimal tPF cut-off at 14.5 IRPC. According to the ROC-calculated cut-off, the number of positive tPF samples increased and showed no significant difference compared to the results of serum samples collected from piglets of either sex (*p* > 0.05; Table 4). The descriptive statistics regarding the test’s results are presented in Table 5. The AUC value for tPF (0.920) was classified as excellent; however, it was significantly lower compared to the reference (*p* < 0.05; Fig. 2). Implementing an optimal cut-off improved the test’s sensitivity, NPV, and accuracy but decreased its PPV and specificity (Table 6). The kappa coefficient increased slightly (from 0.49 to 0.54), maintaining moderate agreement (Table 6).

**Table 4:** The comparison of anti-*E. Rhusiopathiae* antibody presence in different matrices.

Sample type	Positive/Total	Proportion of positive samples (%)	95% CI
Male piglets serum	127/160	79.38^a^	72.45–84.92
Female piglets serum	126/154	81.82^a^	74.98–87.11
tPF (manufacturer’s cut-off)	89/160	55.62^b^	47.88–63.1
tPF (ROC’s cut-off)	127/160	79.38 ^a^	72.45–84.92

^a,b^Different letters represent a statistically significant difference between the analysed samples (*p* < 0.05); 95% CI − 95% confidence interval.

**Table 5:** Mean (± SD) and range (minimum and maximum) IRPC values obtained for *E. rhusiopathiae* ELISA for various samples.

	Mean	Min.	Max.	SD
Male piglets’ serum IRPC	82.58	-3.34	141.69	37.20
Female piglets’ serum IRPC	85.67	-3.03	145.63	35.03
tPF IRPC	57.77	0.80	147.97	41.36

Min. – minimum value; Max. – maximum value; SD – standard deviation.

**Table 6:** The comparison of the selected *E. rhusiopathiae* test parameters and measures of agreement depending on the implemented cut-off values.

Cut-off (IRPC)	40	14.5
Sensitivity	0.70	0.91
Specificity	1	0.64
Positive predictive values	1	0.91
Negative predictive values	0.46	0.64
Accuracy	0.76	0.85
Kappa coefficient	0.49	0.54

Using the cut-off recommended for serum, all low-positive pooled tPF samples, regardless of their dilution, were negative for anti-*E. rhusiopathiae* antibody. However, when the ROC-calculated cut-off was used, all low-positive samples at the dilution of 1:10 and one at the dilution of 1:20 were classified as positive. In total, 0% (0/12) and 33.3% (4/12) of low-positive samples were correctly classified as positive following the manufacturer’s and ROC-calculated cut-off, respectively. Among medium-positive samples, only two were positive for specific antibodies, one at the dilution of 1:10 and one at the dilution of 1:20. Applying the ROC-calculated cut-off value, specific antibodies were detected in all samples at the dilution up to 1:20, and in one sample at the dilution of 1:40. In general, 16.7% (2/12), and 58.3% (7/12) of medium-positive samples were correctly classified using manufacturer’s and ROC-calculated cut-off, respectively. Using the cut-off recommended for serum, specific antibodies were detected in all high-positive samples at the dilution of 1:10 and one sample at the dilution of 1:20. All samples, independently of their dilution, were classified positive when the ROC-calculated cut-off was used; 33.3% (4/12), and 100% (12/12) of high-positive pooled PF samples were correctly classified after using manufacturer’s and ROC-calculated cut-off, respectively.

### HEV

**Fig. 3:**
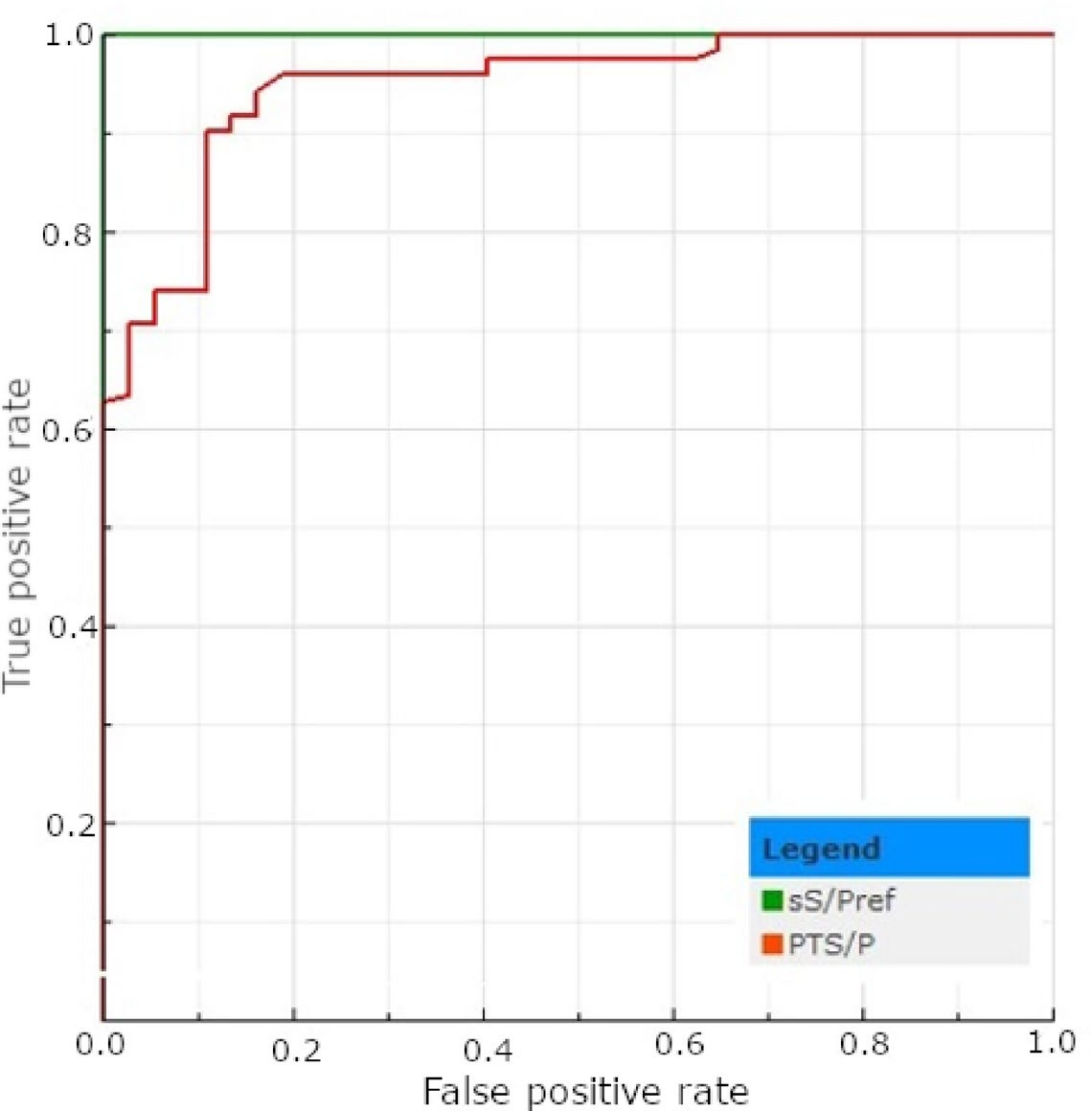
The ROC plots determined for the HEV ELISA test results obtained for serum (sS/Pref) and tPF (PTS/P).

Applying the cut-off value recommended for serum samples (S/P%>65%), 109 out of 160 (68.12%) tPF samples were classified as positive (Table 7). From the corresponding male serum samples, 123 gave positive results (76.88%; Table 7). The number of positive results obtained from female serum was comparable to that of male serum (*p* > 0.05; Table 7). No statistically significant difference has been found between male serum and tPF (*p* > 0.05); however, a significant difference was observed between tPF and female serum (*p* < 0.05; Table 7). Using the ROC-calculated cut-off (S/P%>45%), the number of positive tPF samples for HEV antibodies increased from 109 to 126 (78.75%; Table 7) and was comparable to that of each sex piglet’s serum (*p* > 0.05). The descriptive statistics regarding the test’s results are presented in Table 8. The AUC calculated for tPF (0.947) was classified as excellent, although it was significantly lower than the reference (*p* < 0.05; Fig. 3). Applying the optimal cut-off value increased the test’s sensitivity, NPV, and slightly accuracy but decreased its specificity and PPV (Table 9). The kappa coefficient increased slightly, from 0.75 to 0.76, maintaining the substantial agreement (Table 9).

**Table 7:** The comparison of anti-HEV antibody presence in different matrices.

Sample type	Positive/Total	Proportion of positive samples (%)	95% CI
Male piglets serum	123/160	76.88^a, b^	69.76–82.73
Female piglets serum	116/144	80.56^a^	73.33–86.19
tPF (manufacturer’s cut-off)	109/160	68.12^b^	60.55–74.85
tPF (ROC’s cut-off)	126/160	78.75^a^	71.78–84.38

^a,b^Different letters represent a statistically significant difference between the analysed samples (*p* < 0.05); 95% CI – 95% confidence interval.

**Table 8:** Mean (± SD) and range (minimum and maximum) S/P% values obtained for HEV ELISA for various samples.

	Mean	Min.	Max.	SD
Male piglets’ serum S/P%	144.35	9.00	270.00	68.03
Female piglets’ serum S/P%	144.54	-22.00	276.00	69.90
tPF S/P%	97.78	3.00	267.00	59.23

Min. – minimum value; Max. – maximum value; SD – standard deviation.

**Table 9:** The comparison of the selected HEV test parameters and measures of agreement depending on the implemented cut-off values.

Cut-off (S/*P*%)	65	45
Sensitivity	0.88	0.96
Specificity	0.97	0.78
Positive predictive values	0.99	0.94
Negative predictive values	0.71	0.85
Accuracy	0.9	0.92
Kappa coefficient	0.75	0.76

Following the test manufacturer’s cut-off, all low-positive pooled tPF samples, independent of their dilution, were negative for anti-HEV antibodies. Applying the ROC-calculated cut-off, specific antibodies were detected in only one sample at the dilution of 1:10. Thus, none (0/12) and 8.33% (1/12) out of low-positive samples were correctly classified as positive when the test’s manufacturer and ROC-calculated cut-off values were applied, respectively. Similarly, using the cut-off recommended by the test’s manufacturer, all moderate-positive samples, independent of their dilution, were classified as negative. However, following the ROC-calculated cut-off, specific antibodies were detected in all samples at the dilution of 1:10 and one sample at the dilution of 1:20. In general, none (0/12) and 33% (4/12) of moderate-positive samples were correctly classified, after applying the test’s manufacturer and ROC-calculated cut-off, respectively. According to the cut-off recommended by the test’s manufacturer, specific antibodies were detected in three high-positive samples, two at the dilution of 1:10 and one at the dilution of 1:20. Using the ROC-calculated cut-off, all samples at the dilution up to 1:20, two samples at the dilution of 1:40, and one at the dilution of 1:80 were classified positive. In total, 25% (3/12) and 75% (9/12) of high-positive samples were classified as positive after implementing the test’s manufacturer and ROC-calculated cut-off values, respectively.

### PEDV

**Fig. 4:**
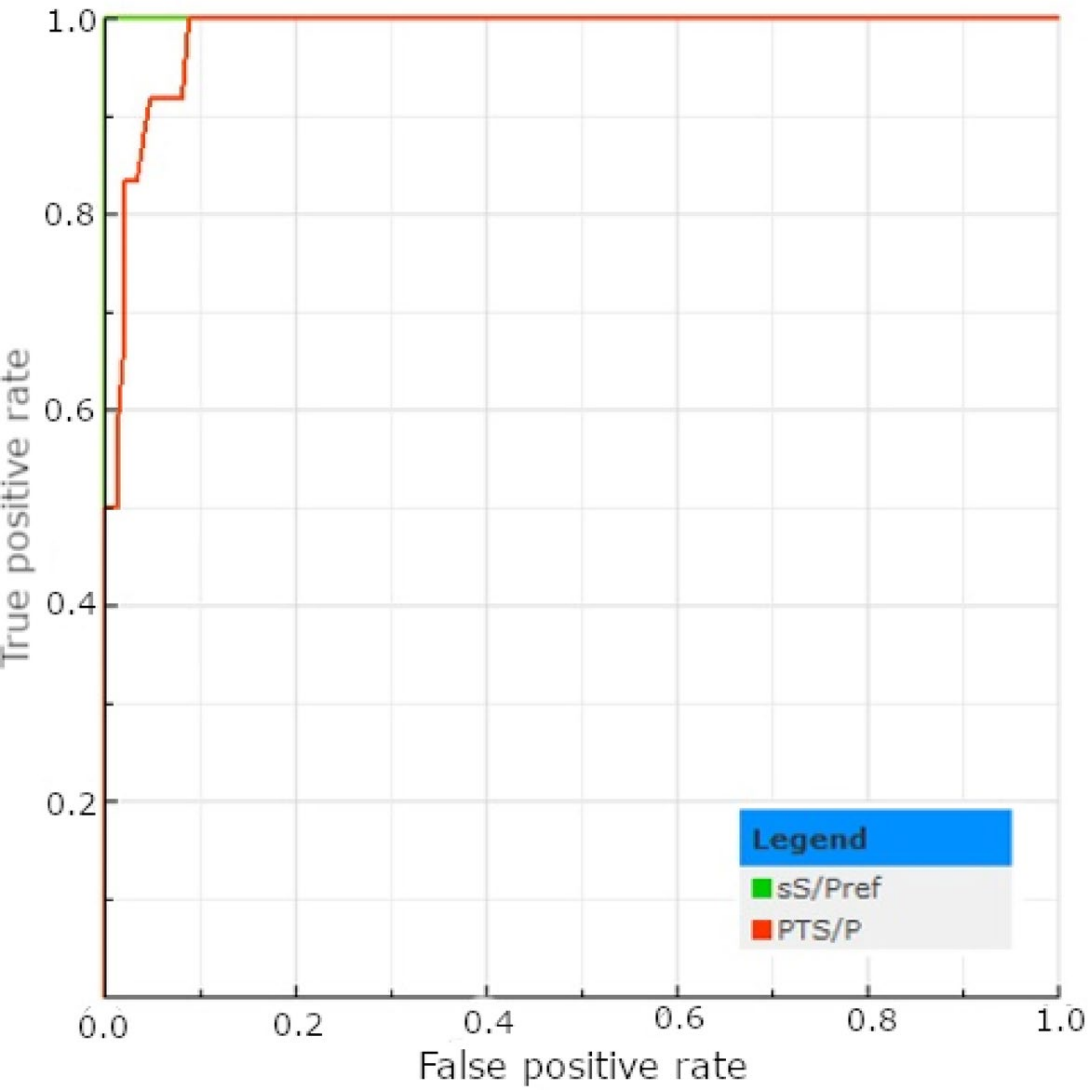
The ROC plots determined for the PEDV ELISA test results obtained for serum (sS/Pref) and tPF (PTS/P).

Following the cut-off recommended by the ELISA test’s manufacturer (S/P%≥60%), 5 out of 160 tPF (3.12%) samples and 12 (7.5%) out of the corresponding male serum samples were positive (Table 10). The number of positive samples between each tested sample type was comparable (*p* > 0.05; Table 10). The ROC curve determined a new cut-off at S/P%>24%, which increased the number of tPF-positive samples from 5 to 13 (8.13%; Table 10). The descriptive statistics regarding the test’s results are presented in Table 11. There was no statistically significant difference between the referenced and tPF’s (0.984) AUC (*p* > 0.05; Fig. 4), which was excellent. Applying the new cut-off increased the test’s sensitivity, NPV, and accuracy while decreasing its specificity and PPV (Table 12). In addition, the increase in the kappa coefficient from 0.57 to 0.78 allows for reclassifying the agreement from moderate to substantial (Table 12).

**Table 10:** The comparison of anti-PEDV antibody presence in different matrices.

Sample type	Positive/Total	Proportion of positive samples (%)	95% CI
Male piglets’ serum	12/160	7.5%	4.34–12.65
Female piglets’ serum	12/146	8.22%	4.76–13.82
tPF (manufacturer’s cut-off)	5/160	3.12%	1.34–7.11
tPF (ROC’s cut-off)	13/160	8.13%	4.81–13.4

**Table 11:** Mean (± SD) and range (minimum and maximum) S/P% values obtained for PEDV ELISA for various samples.

	Mean	Min.	Max.	SD
Male piglets’ serum S/P%	17.94	1.00	114.00	24.01
Female piglets’ serum S/P%	17.05	1.00	120.00	23.01
tPF S/P%	9.23	1.00	81.00	13.61

Min. – minimum value; Max. – maximum value; SD – standard deviation.

**Table 12:** The comparison of the selected PEDV test parameters and measures of agreement depending on the implemented cut-off values.

Cut-off (S/*P*%)	60	24
Sensitivity	0.42	0.83
Specificity	1	0.97
Positive predictive values	1	0.77
Negative predictive values	0.95	0.99
Accuracy	0.96	0.97
Kappa coefficient	0.57	0.78

Because of the low number of positive samples, categorisation regarding antibody presence was impossible; therefore, the influence of the dilution on antibody detection in the pooled tPF sample was not evaluated for PEDV.

### IAV

**Fig. 5:**
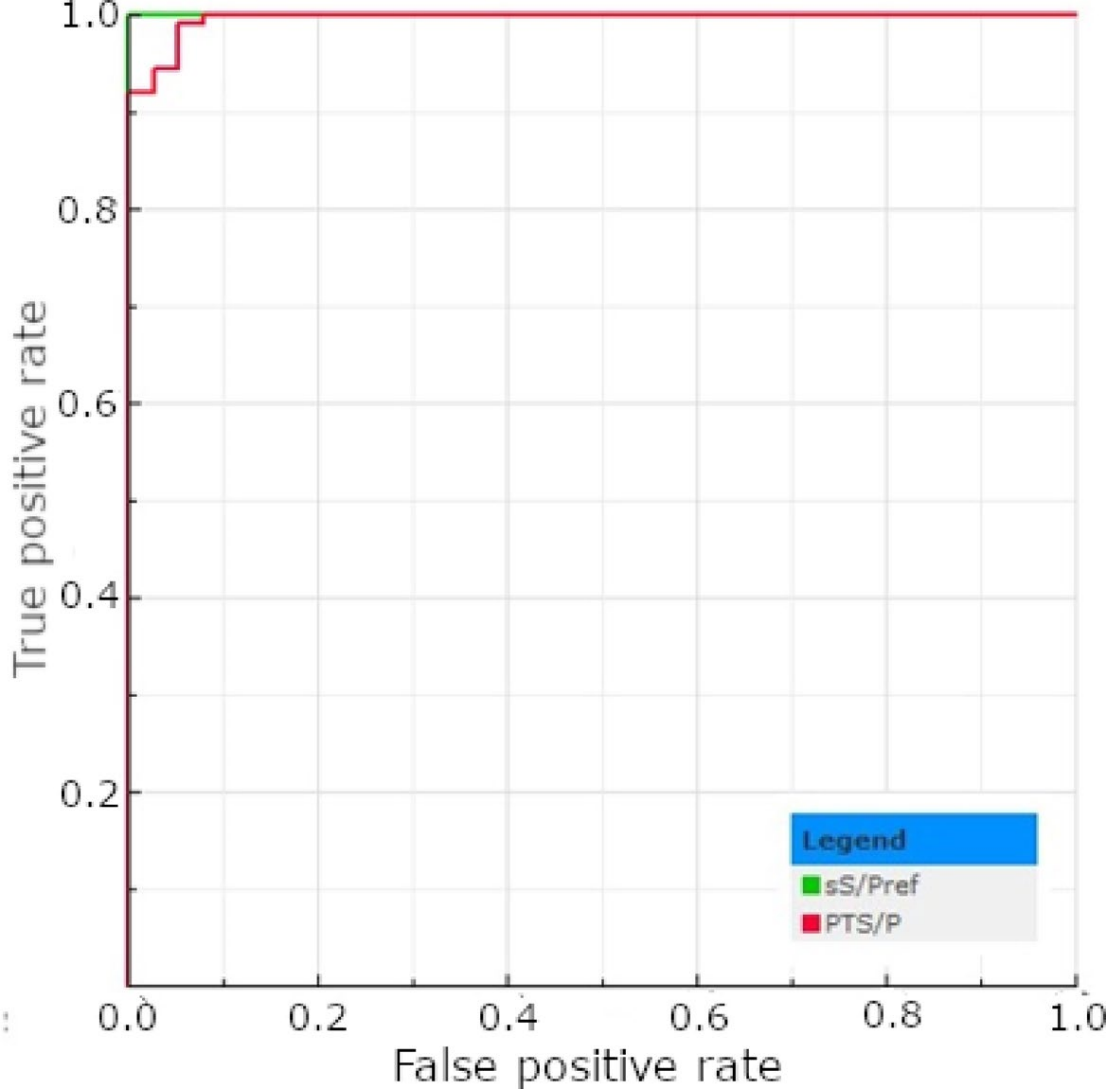
The ROC plots determined for the IAV ELISA test results obtained for serum (sS/Pref) and tPF (PTS/P).

Following the cut-off value recommended by the ELISA test manufacturer (S/N%<47%), 82 out of 160 PF (51.25%) samples were classified as positive (Table 13). From the corresponding sera, 124 (77.5%) samples gave positive results (Table 13). The number of positive samples was comparable between male and female serum (*p* > 0.05; Table 13); however, the number of positive tPF was significantly lower than the positive number of each sex piglet’s sera (*p* < 0.05; Table 13). Using the ROC curve, an optimal cut-off value at 83 S/N% was determined for tPF and increased the number of tPF samples classified as positive to 125 (78.12%; Table 13), which was comparable to the results obtained from serum samples of each sex piglet (*p* > 0.05; Table 13). The descriptive statistics regarding the test’s results are presented in Table 14. There was no statistically significant difference between the references and tPF’s (0.996) AUC (*p* > 0.05; Fig. 5), which was excellent. Applying the ROC-calculated cut-off allowed for an increase in the test’s sensitivity, NPV, and accuracy. Meanwhile, the decrease in its specificity and PPV was only slight (Table 15). Implementing a new cut-off resulted in considerable growth of the kappa coefficient from 0.47 to 0.93, improving the agreement from moderate to great (Table 15).

**Table 13:** The comparison of anti-IAV antibody presence in different matrices.

Sample type	Positive/Total	Proportion of positive samples (%)	95% CI
Male piglets serum	124/160	77.5%^a^	70.43–83.28
Female piglets serum	129/163	79.14%^a^	72.27–84.67
tPF (manufacturer’s cut-off)	82/160	51.25%^b^	43.57–58.87
tPF (ROC’s cut-off)	125/160	78.12%^a^	71.1-83.83

^a,b^Different letters represent a statistically significant difference between the analysed samples (*p* < 0.05) 95% CI – 95% confidence interval.

**Table 14:** Mean (± SD) and range (minimum and maximum ) S/N% values obtained for IAV ELISA for various samples.

	Mean	Min.	Max.	SD
Male piglets’ serum S/N%	29.65	2.97	106.59	32.71
Female piglets’ serum S/N%	27.64	3.06	105.14	31.14
tPF S/N%	49.47	3.66	104.86	31.88

Min. – minimum value; Max. – maximum value; SD – standard deviation.

**Table 15:** The comparison of the selected IAV test parameters and measures of agreement depending on the implemented cut-off values.

Cut-off (S/*N*%)	47	83
Sensitivity	0.66	0.99
Specificity	1	0.92
Positive predictive values	1	0.98
Negative predictive values	0.46	0.97
Accuracy	0.74	0.98
Kappa coefficient	0.47	0.93

Applying the test manufacturer’s cut-off value, all low-positive samples, regardless of their dilution, were classified as negative for the presence of anti-IAV antibodies. Respecting the ROC-calculated cut-off, two out of three low-positive samples at the dilution 1:10 were classified as positive. Hence, none (0/12) and 16.7% (2/12) of low-positive samples were correctly classified as positive, following the manufacturer’s and ROC-calculated cut-off, respectively. All moderate-positive samples, independent of dilution, were negative to the specific antibody when classified according to the test manufacturer’s cut-off. However, when the ROC-calculated cut-off was applied, all moderate-positive samples at the dilution of 1:10, two at the dilution of 1:20, and two at the dilution of 1:40 were classified as positive. None (0/12) and 58.3% (7/12) of moderate-positive samples were correctly classified as positive after using the test’s manufacturer or ROC-calculated cut-offs, respectively. Using the test’s manufacturer cut-off, specific antibodies were detected in all high-positive samples at the dilution of 1:10. Respecting the ROC-calculated cut-off value, all high-positive samples at the dilution of up to 1:40 and two at the dilution of 1:80 were classified as positive. In general, 25% (3/12) and 91.7% (11/12) of high-positive samples were correctly classified as positive after applying the manufacturer’s or ROC-calculated cut-off, respectively.

### *M. hyopneumoniae*

**Fig. 6:**
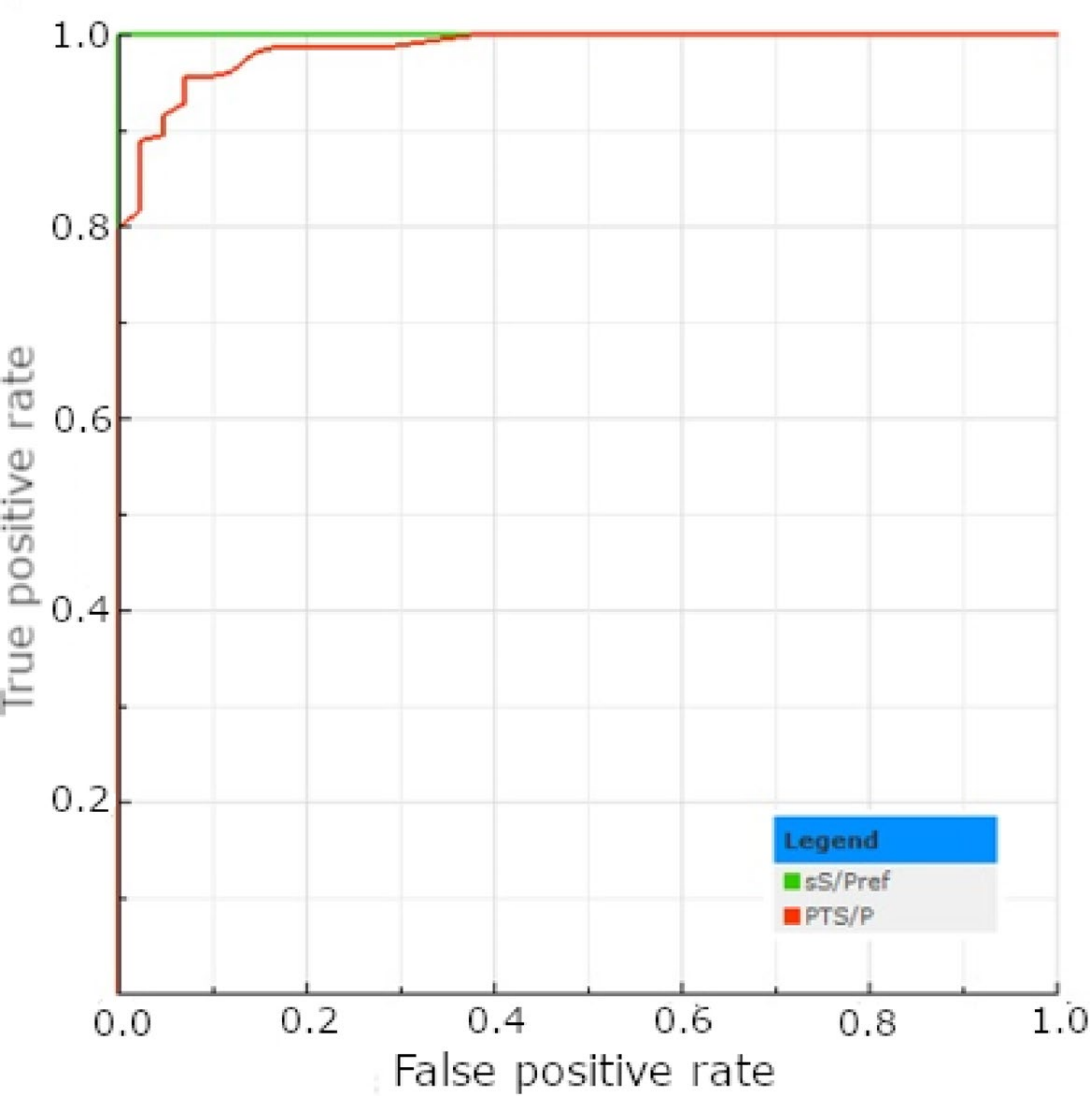
The ROC plots determined for the *M. hyopneumoniae* ELISA test results obtained for serum (sS/Pref) and tPF (PTS/P).

Using the cut-off recommended for serum (S/*P* > 0.35), 105 out of 196 tPF (53.57%) samples and 154 (78.57%) out of the corresponding male serum samples were classified as positive (Table 16). The number of positive female piglet sera was comparable to that of positive male sera (*p* > 0.05; Table 16). The positive results from the tPF samples were significantly lower than those obtained from male and female sera (*p* < 0.05; Table 16). Using the ROC-calculated cut-off (S/*P* > 0.05), the number of positive tPF samples increased to 159 (81.12%; Table 16), which was comparable to the positive sera number of piglets belonging to both genders (*p* > 0.05). The descriptive statistics regarding the test’s results are presented in Table 17. The AUC determined for tPF (0.986) was significantly lower compared to the reference (*p* < 0.05; Fig. 6); however, it was classified as excellent. Implementing the new cut-off increased the values of almost all the test’s parameters, except specificity, which was only slightly decreased, and the measure of agreement (kappa coefficient increased from 0.37 to 86), which was reclassified from fair to great (Table 18).

**Table 16:** The comparison of anti-*M. Hyopneumoniae* antibody presence in different matrices.

Sample type	Positive/Total	Proportion of positive samples (%)	95% CI
Male piglets serum	154/196	78.57%^a^	72.31–83.74
Female piglets serum	156/209	74.64%^a^	68.33–80.06
tPF (manufacturer’s cut-off)	105/196	53.57%^b^	46.59–60.42
tPF (ROC’s cut-off)	159/196	81.12%^a^	75.07–85.98

^a,b^Different letters represent a statistically significant difference between the analysed samples (*p* < 0.05) 95% CI – 95% confidence interval.

**Table 17:** Mean (± SD) and range (minimum and maximum) S/P values obtained for *M. hyopenumoanie* ELISA for various samples.

	Mean	Min.	Max.	SD
Male piglets’ serum S/P	0.78	-0.05	1.91	0.49
Female piglets’ serum S/P	0.77	-0.04	2.26	0.53
tPF S/P	0.40	-0.06	2.03	0.40

Min. – minimum value; Max. – maximum value; SD – standard deviation.

**Table 18:** The comparison of the selected *M. hyopneumoniae* test parameters and measures of agreement depending on the implemented cut-off values.

Cut-off (S/*P*)	0.35	0.05
Sensitivity	0.65	0.99
Specificity	0.88	0.83
Positive predictive values	0.95	0.96
Negative predictive values	0.41	0.95
Accuracy	0.70	0.95
Kappa coefficient	0.37	0.86

Applying the cut-off value recommended by the test’s manufacturer, all low-positive samples, regardless of the dilution, were negative for the anti-*M.hyopneumoniae* antibodies. When samples were classified using ROC-calculated cut-off, specific antibodies were detected in two out of three samples at the dilution of 1:10. Thus, none (0/12), and 16.7% (2/12) of low-positive samples were correctly classified as positive, after using the test’s manufacturer and ROC-calculated cut-off. Using the manufacturer’s cut-off, all moderate-positive samples were classified as negative. However, when applying the ROC-calculated cut-off, specific antibodies were detected in all samples at the dilutions up to 1:40. Thus, none (0/12) and 75% (9/12) of moderate-positive samples were correctly classified as positive following the test’s manufacturer’s ROC-calculated cut-off, respectively. Using the test’s manufacturer’s cut-off, specific antibodies were detected in all high-positive samples at the dilution of 1:10; nevertheless, when applying an ROC-calculated cut-off, all high-positive samples were classified as positive. In total, 25% (3/12) and 100% (12/12) of high-positive samples were correctly classified following the test manufacturer’s or ROC-calculated cut-offs, respectively.

## Discussion

Despite being recognised for approximately 15 years as a promising diagnostic matrix for surveillance and disease monitoring in pigs, PF remains underexplored compared to other matrices. Consequently, there is a lack of commercially dedicated and validated diagnostic tests for PF. This scarcity necessitates using kits designed for other sample types, e.g., serum. Commercial ELISA kits are typically validated using serum samples collected from animals with well-defined health status, such as those clinically infected or known to be seronegative. Therefore, the cut-off values recommended by the manufacturers are usually specific to both the sample type and the disease context applied during test development. Applying these cut-offs to alternative matrices, such as PF or MJ, which may differ in composition and antibody concentration, may lead to suboptimal test performance. Much like MJ, PF contains both serum, lymph, and intracellular fluid, which can dilute the serum content^16^. Available data suggest that antibody concentration in other biological fluids, such as mentioned MJ, is lower than in serum^16^. However, the referenced study also shows a high agreement between ELISA results of serum and MJ samples when adequate dilutions were implemented and excellent sensitivity and specificity for MJ, which increased upon implementing different cut-offs^16^. As shown in the present study, several test parameters—such as sensitivity, NPV, and the kappa coefficient—were unsatisfactory when the manufacturer’s cut-off values were used for tPF samples. However, calculating and implementing matrix-specific optimal cut-offs using ROC analysis considerably improved the performance of most evaluated ELISA kits. These findings underscore the importance of verifying and, if needed, recalibrating diagnostic thresholds when commercial ELISA kits are applied to sample types or testing conditions different from those for which they were initially designed.

The ideal diagnostic test should exhibit high values across all performance parameters; however, achieving this balance is often challenging. Sensitivity and specificity are measures of a diagnostic test’s ability to correctly classify an animal as having or not having a disease. Sensitivity describes the ability to designate an animal with a disease as positive; if the diagnostic test is highly sensitive, it has few false-negative results, so fewer positive cases are missed. Specificity refers to the ability to identify an animal that does not have a disease as negative; if a test has high specificity, there are fewer false-positive results. It is ideal to use a test with high sensitivity and high specificity. Improving one parameter often leads to a decline in another. Depending on their purpose, diagnostic tests can be classified into discovery (screening), confirmatory, and exclusion tests^17^.

A good screening test should provide accurate results, so choosing tests with high sensitivity and specificity is reasonable. Positive results should be interpreted cautiously when a screening test is used in a generally healthy population. Confirmatory testing is often necessary to distinguish between true positive and false positive results obtained on initial screening^18,19^. Understanding the predictive value when interpreting results obtained with a particular test in a specific population is important. PPV and NPV values depend on the sensitivity and specificity of the test being used and on the prevalence of the disease in the population. Predictive values can be regarded as ‘reliable’ results of individual tests. If the PPV is high, positive test results are generally accurate, whereas negative results may be questioned. If the disease is rare in the population, the positive predictive value will be low, regardless of the accuracy of the test. If there is a high NPV, negative test results are generally accurate, while positive results may be questioned^18,20^. Determining and applying the optimal cut-off for tPF indicates the potential utility of some commercial ELISA tests for this sample.

For example, regarding the *M. hyopneumoniae* ELISA kit, implementing the optimal tPF’s cut-off considerably improved almost all analysed performance parameters to near-perfect values, resulting in excellent accuracy and great agreement. The AUC value calculated for tPF, despite being significantly lower than for serum, was excellent, indicating collectively that the evaluated kit represents a useful tool for detecting specific antibodies in tPF.

A similar scenario was observed with the IAV kit. Implementing the ROC-calculated tPF cut-off improved previously unsatisfactory performance parameters, only slightly decreasing others, which gave excellent accuracy and great agreement. Alongside the comparable AUC for these matrices, this indicates the usefulness of the evaluated test for assessing the presence of specific antibodies in tPF after determining the optimal cut-off.

The PEDV kit displayed excellent accuracy regarding tPF even when respecting the cut-off recommended by the test’s manufacturer; however, the proportion of anti-PEDV positive samples independent of the sample type was low, and it is known that diagnostic accuracy depends on prevalence^21^. The new cut-off considerably increased previously unsatisfactory sensitivity and the agreement, which was still acceptable despite being lower than in the case of the two above-described kits. These data, collectively with the comparable AUC values for tPF and the reference, indicate this kit’s utility in detecting specific antibodies in tPF following the implementation of optimal cut-off.

Regarding the HEV ELISA kit, implementing the optimal tPF cut-off drove only a slight increase in the agreement, which did not change its classification and caused a considerable decrease in specificity, indicating the possibility of giving false positive results. Despite the AUC for tPF being significantly lower than the reference, it was classified as excellent, collectively with excellent sensitivity, accuracy, and acceptable remaining performance parameters, indicating this kit’s possible utility in specific antibody detection in tPF following the new cut-off implementation.

Less promising results were obtained regarding the two remaining kits. The AUC value for tPF in the *E. rhusiopathiae* kit was classified as excellent despite being significantly lower than the reference. The optimal cut-off increased some of the analysed performance parameters of the *E. rhusiopathiae* kit, considerably decreasing specificity and indicating the risk of false-positive results. What is more, the increase in NPV was insufficient. Therefore, some performance parameters remained unsatisfactory despite the implementation of the optimal cut-off for the tPF. Collectively, with a fair agreement, this questioned the kit’s utility in assessing the presence of specific antibodies in tPF.

Although the optimal cut-off improved some of the *A. pleuropneumoniae* kit’s parameters, the obtained sensitivity and NPV were the lowest among all kits evaluated in the present study. Moreover, it decreased specificity considerably and did not change the agreement classification, leaving it moderate only. Alongside the considerable AUC, this suggests the limited utility of this kit for detecting specific antibodies in tPF.

As mentioned previously, a decreasing trend in piglets’ tail docking is observed in some countries^15^. In the European Union, the routine performance of this procedure is prohibited by the law restrictions^22^. Under such conditions, PF samples are collected from male piglets only. This may raise questions about the suitability of tPF for detecting specific indices in piglets of both genders. Thus, in the present study, the utility of PF obtained exclusively from testicles for detecting the presence of specific antibodies in male and female piglets was assessed. For this purpose, the differences in the proportion of positive samples between the serum of male piglets, female piglets, and tPF were determined. The present results indicate that the frequency of detected antibodies in tPF can be extrapolated to females. Thus, tPF can serve as an alternative sample to the serum of piglets belonging to both genders for detecting antibodies against major pig pathogens circulating in the herd.

However, this utility depends mainly on the ELISA kit used and the applied cut-off. For example, concerning the PEDV ELISA kit, there was no statistically significant difference in the proportion of positive samples among all tested matrices, regardless of the applied cut-off. In the case of the HEV ELISA kit, significant differences in the number of positive samples were observed between the serum of female piglets and tPF only; however, after implementing the ROC-calculated cut-off for tPF, these values were comparable.

On the other hand, regarding the *M. hyopneumoniae*,* E. rhusiopathiae*, and IAV ELISA kits, such differences were demonstrated between the serum of piglets belonging to both genders and tPF classified according to the test manufacturer’s cut-off. When the ROC-calculated cut-off was used, these differences disappeared. It highlights the utility of tPF for specific antibody detection when using the optimal cut-off for tPF. However, regarding the APP ELISA kit, establishing and applying the optimal tPF cut-off did not eliminate the differences in the number of positive samples between piglets’ serum and tPF, thereby limiting the utility of tPF for this ELISA kit.

Existing literature on the topic addressed in this study is scarce. One of the studies highlighted the potential utility of a commercial ELISA kit for monitoring anti-*M. hyopneumoniae* antibodies in breeding herds^12^. The test used in the abovementioned study demonstrated excellent diagnostic sensitivity and specificity, at 97.6% and 100%, respectively; additionally, implementing an optimal cut-off for PF increased the first of the parameters^12^. Another study demonstrated the suitability of a commercial ELISA kit, with slight procedural modifications, for detecting specific anti-PRRSV IgG in PF. Depending on the cut-off value applied, the kit achieved up to 99.0% sensitivity and 100% specificity^8^. The study’s authors considered PF a cost-saving material for PRRSV monitoring in the negative or naïve breeding herds^8^. In both studies, lower dilutions of PF were used than those recommended for serum, and the tested PF represented pooled samples^8,12^. In addition, in the study by Lopez et al. (2022), selected test components, like conjugate or incubation time, were modified, which could influence the obtained results^8^. Such an approach generates additional work and costs. Herein, our results provide the data regarding the utility of commercial ELISA kits to detect the presence of specific antibodies in tPF without any modification in the test procedure and the usefulness of individual tPF sample testing. In the study conducted by Di Bartolo et al. that aimed to assess individual PF samples’ utility for specific anti-HEV antibody detection, respecting the cut-off recommended for serum samples, next to the good specificity (89%), the unsatisfying sensitivity (0.47) and slight agreement (0.19) were observed^14^. Applying the cut-off determined by ROC analysis results in 77% sensitivity and 56% specificity, with an increase in agreement to a fair level (0.27). Interestingly, the sensitivity and specificity were 88% and 67% at the litter level, respectively, and the agreement was moderated^14^.

Most available studies evaluating PF utility used aggregate PF samples rather than individual PF. Under field conditions, pooled sample testing can sometimes be more favourable than testing individual samples, such as in pathogen surveillance. However, pooling can affect the test results by diluting the sample. The obtained data emphasise the importance of calculating and applying an optimal cut-off for matrices other than those evaluated. Applying the ROC-calculated cut-off considerably increased the number of correctly classified pooled samples regarding each test. The present results showed, however, that pooling can influence the obtained results. The maximum dilution in which the samples were correctly classified depended on the sample positivity level. Regarding high-positive samples, 4 out of 5 evaluated ELISA kits correctly classified 100% of positive samples of the dilution up to 1:40, and 2 of them correctly classified all positive samples in each evaluated dilution when the ROC-calculated cut-off was applied. Among moderate-positive samples, only 2 ELISA kits correctly classified all samples in the dilution up to 1:40, when the ROC-calculated cut-off was applied. In the case of low-positive samples, 3 out of the evaluated ELISA kits failed to distinguish all positive samples at the dilution of 1:10. However, one ELISA kit correctly classified all samples in the dilution up to 1:20, and one sample in the dilution of 1:40, using the ROC-calculated cut-off. Using the tests’ manufacturers’ cut-offs, most tests did not correctly classify any low-positive and/or moderate-positive samples, regardless of the dilution. Our results align with the findings of the study by Di Bartolo et al. (2020), who also evaluated the effect of dilution on different PF samples’ positivity levels^14^. Strong, medium, medium-weak, and weak ELISA samples diluted to 1:4, 1:5, 1:6, and 1:7 were tested with the commercial ELISA kit dedicated to serum. Using the ROC-calculated cut-off, samples of all levels, independent of dilution, were correctly classified. When applying the cut-off recommended by the test’s manufacturer, only strong positive samples at all dilutions were correctly classified. Meanwhile, the test failed to classify weak samples correctly^14^.

In summary, the results of the current study suggest that specific commercially available ELISA kits, initially designed for detecting specific antibodies in serum, can also be used to identify these antibodies in tPF, provided that the optimal cut-off is applied. However, this highly depends on the specific ELISA kit used; thus, individual analysis is necessary to determine the optimal cut-off value for each sample type. Additionally, each test’s performance parameters and measures of agreement should be assessed. The test’s parameters and agreement measures remained unsatisfactory for some kits, even when the optimal tPF cut-off was applied. These tests may require procedural modifications before being used for tPF analysis. When tPF was tested as a pooled sample, applying the modified cut-off provided significantly better discrimination of positive samples than the serum-specific cut-off. However, some samples, particularly those with high dilution, were still misclassified. The lack of significant differences between the number of positive samples detected in male and female serum and between the serum of both sexes and tPF indicates the potential utility of tPF for assessing the presence of specific antibodies in whole litters.

## Methods

### Samples

Samples used in the present study were collected from 6 commercial herds across Poland, though accurate health status data were not available for most of them. The samples were collected by veterinarians for various diagnostic purposes. tPF was obtained from testes removed by veterinarians during routine castrations of boars, which were carried out as part of standard animal husbandry practices in these herds. Consequently, no additional experimental procedures were implemented for animal sample collection. Blood was taken from males and females from the *vena cava cranialis* into clot activator tubes. tPF was recovered from testicles collected into falcon tubes during boar castration. All samples were transported to a laboratory in cooling conditions. Blood was centrifuged at 2500 × g for 15 min at 4 °C. Subsequently, all obtained serum samples were kept at -70 °C until analyses.

### Laboratory analyses

Applying commercially available ELISA kits validated for serum, collected samples were evaluated for the presence of antibodies against six pathogens: *A. pleuropneumoniae* (ID Screen APP Screening Indirect (serotypes 1 through 12), ID vet, Grables, France), *E. rhusiopathiae* (Civtest suis SE/MR, Hipra, Amer, Spain), HEV (ID Screen Hepatitis E Indirect Multi-species, ID vet, Grables, France), PEDV (ID Screen PEDV Indirect; ID vet, Grables, France), IAV (ID Screen Influenza A Antibody Competition Multi-Speecies; ID vet, Grables, France), and *M. hyopneumonaie* (*Mycoplasma hyopneumoniae* Antibody Test Kit, Idexx, Westbrook, USA). The number of samples subjected to each analysis is presented in Table 19. Each test was performed, and the results were expressed according to the manufacturer’s recommendations. The optical density (OD) was measured via Magellan v.7.2 software (Tecan), and the results were calculated according to the formulas recommended by the manufacturers of the kits. For *A. pleuropneumoniae* and PEDV kits, OD values were transformed to sample to positive (S/P) ratio calculated upon the following formula: S/P% = (sample’s OD – negative control’s OD)/(positive control’s OD – negative control’s OD) × 100. For the *M. hyopneumoniae* kit, OD values were transformed to S/P ratio according to this formula: S/P = (sample’s OD – negative control’s OD)/(positive control’s OD – negative control’s OD). For the *E. rhusiopathiae* kit, the OD values were transformed to IRPC (Relative Index × 100), calculated as follows: IRPC = (sample’s OD – negative control’s OD)/(positive control’s OD-negative control’s OD) × 10. The HEV kit was a bi-well test, meaning each sample and control must be deposited in duplicate. For each sample, a ned OD value had to be determined by calculating the difference between even wells’ OD and odd wells’ OD. Subsequently, the results were transformed to S/P% upon the following formula: S/P% = sample’s ned OD/positive control’s net OD × 100. For the IAV kit, OD values were transformed to a sample-to-negative (S/N) ratio calculated as follows: S/N% = sample’s OD/negative control’s OD × 100.

**Table 19:** The number of samples subjected to each laboratory analysis.

Assay direction	Male piglets serum	Female piglets serum	tPF	Total
*A. pleuropneumoniae*	178	159	178	515
*E. rhusiopathiae*	160	154	160	474
*M. hyopneumoniae*	196	209	196	601
IAV	160	163	160	483
HEV	160	144	160	464
PEDV	160	146	160	466

To assess the impact of pooling on the test results, the samples were categorised as low, moderate, and high-positives, according to the ranges presented in Table 20. The ranges provided are based on the distribution of results observed in this study and should be interpreted with caution, as they may not precisely reflect the antibody concentrations in individual samples.

**Table 20:** The classification ranges used for grouping samples as low-, intermediate-, or high-positive.

Assay direction	Ranges	Low-positive	Moderate-positive	High-positive
*A. pleuropneumoniae*	S/P%	≤ 70	> 70 ≤ 180	> 180
*E. rhusiopathiae*	IRPC	≤ 60	> 60 ≤ 100	> 100
*M. hyopneumoanie*	S/P	≥ 1.0	< 1.0 ≥ 0.5	< 0.5
IAV	S/N%	≥ 40	< 40 ≥15	< 15
HEV	S/P%	< 100	≥ 100 < 150	≥ 150
PEDV	S/P%	Not performed		

Three positive samples were randomly selected for each range. Subsequently, for the pooling simulation, 10 µl of tPF from each range (low, moderate, and high-positive) were combined with appropriate volumes of negative tPF sample to mimic pools of 10, 20, 40, and 80 samples, each containing 1 positive sample and 9, 19, 39, and 79 negative samples, respectively. In total, 9 samples for each ELISA kit were assessed in four dilutions. The pooled samples were then tested according to the kit’s instructions.

### Statistical analyses

In the present study, the results of serum sample analysis represent a reference method. A commercial software package was used to calculate the proportion of positive samples for each tested matrix type (Statistica 13.3, TIBCO). The Wilson score method was used to calculate 95% confidence intervals. Differences in the proportion of antibody presence between the tested matrices were assessed using a chi-square test. The StAR software was used to generate and analyse the ROC curves to calculate the optimal tPF cut-offs and compare the area under the curve (AUC) for tPF and serum. Obtained AUC values were classified as follows: 0.9 ≤ AUC – excellent; 0.8 ≤ AUC < 0.9 – considerable; 0.7 ≤ AUC < 0.8 – fair; 0.6 ≤ AUC < 0.7 – poor; 0.5 ≤ AUC < 0.6 – fail^23^. The tests’ performance parameters, including sensitivity, specificity, positive predictive value (PPV), negative predictive value (NPV), and accuracy, were calculated for both, recommended by the tests’ manufacturers and ROC-calculated cut-offs. The agreement between the results obtained from serum samples and tPF was assessed with the kappa coefficient and was evaluated according to the following classification: < 0.2 – poor; 0.2–0.4 – fair; 0.41–0.6 – moderate; 0.61-0.8-substantial; >0.8 – great^24^. For all tests, the p-value was considered significant at < 0.05.

Ethics declarations: According to the Act on the Protection of Animals Used for Scientific or Educational Purposes in Poland adopted on 15th January 2015 and according to earlier regulations (Act on the Protection of Animals Used for Scientific or Educational Purposes in Poland adopted on 21st January 2005), the study described in this manuscript did not require the permission of the Local Ethical Commission for Investigations on Animals as all samples were collected during standard healthcare procedures implemented by veterinarians caring for the herds to routinely monitor the health status of the herds selected for this study (i.e. serological profiles). The testicular-only processing fluid was obtained from the testis removed during routine boar castrations implemented in those herds. Thus, no extra experimental procedures were applied to collect samples from animals. Informed consent was obtained from the owner to use the animals in the study. All methods were carried out by relevant guidelines and regulations. All methods are reported in accordance with ARRIVE guidelines.

## Data Availability

The datasets used and/or analysed during the current study available from the corresponding author on reasonable request.
